# DHA and EPA exacerbate hypoxia-induced ferroptosis in gastric and small intestinal mucosa by disrupting the balance between SLC7A11 upregulation and PUFA-PL accumulation

**DOI:** 10.1016/j.jlr.2025.100876

**Published:** 2025-08-12

**Authors:** Zhenmei Song, Xuexin Wang, Jie Zeng, Fangli Ren, Yinyin Wang, Meng Li, Qing Lin, Wenli Li, Xingchen Liao, Dezhi Wang

**Affiliations:** 1 Department of Gastroenterology, China-Japan Friendship Hospital, Beijing, China; 2 Medical School of Chinese PLA, Chinese PLA General Hospital, Beijing, China; 3 Department of Gastroenterology, The Seventh Medical Center of Chinese PLA General Hospital, Beijing, China; 4 Department of Urology, The Second Affiliated Hospital, School of Medicine, South China University of Technology, Guangzhou, China; 5 State Key Laboratory of Membrane Biology, School of Medicine, Institute of Precision Medicine, Tsinghua University, Beijing, China; 6 Department of Anesthesiology and Critical Care Medicine, School of Medicine, Johns Hopkins University, Baltimore, USA; 7 Department of Rheumatology, China-Japan Friendship Hospital, Beijing, China

**Keywords:** DHA, EPA, PUFA-PLs, Ferroptosis, Hypoxia, SLC7A11, Gastrointestinal mucosa

## Abstract

Hypoxia, resulting from environmental factors or diseases, can disrupt the gastrointestinal mucosal barrier. Our previous study demonstrated that hypoxia induced ferroptosis in the gastric and small intestinal mucosa by upregulating ALOX5, NOX4, and polyunsaturated fatty acid-containing phospholipids (PUFA-PLs). The impact of docosahexaenoic acid (DHA) and eicosapentaenoic acid (EPA) on ferroptosis is currently a subject of debate. While DHA and EPA upregulate SLC7A11 expression, mitigating lipid peroxidation, they also elevate PUFA-PL levels, exacerbating it. This study investigated the effects and underlying mechanisms of DHA and EPA supplementation on gastric and small intestinal mucosal ferroptosis under normoxic and hypoxic conditions in vitro and in vivo. Under normoxia, DHA and EPA upregulated SLC7A11 expression through the cAMP/PKA/ATF3 pathway, thereby enhancing cellular resistance to lipid peroxidation associated with increased PUFA-PL levels and preventing ferroptosis. In contrast, under hypoxia, DHA and EPA exacerbated ferroptosis by further increasing PUFA-PL levels, which, in combination with hypoxia-induced ALOX5 and NOX4 expression, resulted in excessive lipid peroxidation that overwhelmed the protective mechanisms mediated by SLC7A11 upregulation. These findings indicate that DHA and EPA exacerbate hypoxia-induced ferroptosis in gastric and small intestinal mucosa. Therefore, individuals at risk of hypoxia should carefully consider the potential risks associated with DHA and EPA intake.

## Graphical Abstract

**Figure:**
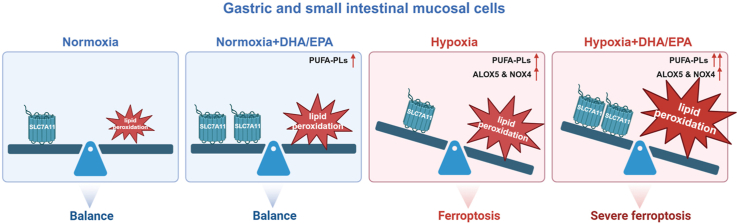

Omega-3 [(n-3)] polyunsaturated fatty acids (PUFAs) are a class of long-chain fatty acids with significant physiological functions. Among these n-3 PUFAs, eicosapentaenoic acid (EPA; 20:5n–3) and docosahexaenoic acid (DHA; 22:6n–3), primarily derived from fish oil, have been extensively studied for their health benefits (1), including hypotriglyceridemic, insulin-sensitizing, anticancer, antioxidant, antidepressant, anti-aging, and anti-inflammatory effects (2). Consequently, DHA and EPA supplementation is widely recommended for general health, with daily intake guidelines typically ranging from 250 to 500 mg, depending on national and regional recommendations.

Ferroptosis is a form of regulated cell death characterized by excessive iron-dependent peroxidation of PUFA-containing phospholipids (PUFA-PLs) within the cell membrane. Under oxidative stress, elevated PUFA levels promote excessive lipid peroxidation, leading to cellular dysfunction and death (3, 4). Dietary supplementation with DHA and EPA has been implicated in ferroptosis-related pathologies, including inflammatory bowel disease, where small intestinal epithelial ferroptosis contributes to disease progression (5, 6). Additionally, excessive intracellular accumulation of DHA and EPA, beyond the lipid droplet storage capacity, has been shown to induce ferroptosis in various cancer cells (7).

The cystine/glutamate antiporter SLC7A11 (also known as xCT) is a key regulator of ferroptosis. It facilitates cystine uptake for glutathione (GSH) biosynthesis, a critical intracellular antioxidant that neutralizes lipid peroxidation and suppresses ferroptosis through the glutathione peroxidase 4 (GPX4)/GSH axis (8). Studies have shown that DHA and EPA upregulate SLC7A11 mRNA and protein expression (9, 10), suggesting a protective role against ferroptosis.

Hypoxia, a state of reduced oxygen availability, can result from various physiological factors, including decreased cardiac output, anemia, and pulmonary diseases, as well as environmental conditions such as high altitude and confined working spaces (11, 12, 13). Oxidative stress due to hypoxia can disrupt the gastrointestinal mucosal barrier, leading to symptoms such as loss of appetite, nausea, vomiting, abdominal distension and pain, diarrhea, and occult gastrointestinal bleeding (12, 14).

Our previous study identified the gastric and small intestinal mucosa as the primary sites of hypoxia-induced gastrointestinal injury, with ferroptosis as a key underlying mechanism. Specifically, hypoxia-induced ferroptosis is mediated by hypoxia-inducible factor-α (HIF-α)-mediated arachidonate 5-lipoxygenase (ALOX5), NADPH oxidase 4 (NOX4), and PUFA-PLs (15). ALOXs and reactive oxygen species (ROS), partially generated by NOXs, can oxidize PUFA-PLs (16, 17). Although DHA and EPA have been shown to upregulate SLC7A11 (a ferroptosis suppressor) and PUFA-PLs (a ferroptosis promoter), their overall impact on ferroptosis remains unclear. This contradictory effect raises two critical questions: (1) Do DHA and EPA exert differential effects on the gastrointestinal mucosa under hypoxic vs. normoxic conditions? (2) What are the molecular mechanisms underlying these effects? Given the potential for DHA and EPA to induce ferroptosis, it is essential to determine whether their consumption as dietary supplements may lead to gastrointestinal mucosal injury, particularly in populations exposed to hypoxia.

This study aimed to investigate the role and mechanisms of DHA and EPA in hypoxia-induced ferroptosis in gastric and small intestinal mucosa, providing insights into their safe and effective use in individuals at risk of hypoxia.

## Materials and Methods

### Reagents and chemicals

Dulbecco's modified Eagle’s medium (DMEM), fetal bovine serum (FBS), and penicillin-streptomycin were obtained from Corning. Primary antibodies used in this study included SLC7A11 (ab307601; Abcam), HIF-1α (ab179483; Abcam), activating transcription factor 3 (ATF3; ER1903-99; HUABIO), NOX4 (A22149; ABclonal), ALOX5 (A2877, ABclonal), GPX4 (ab125066; Abcam), Caspase-3 (A19654, ABclonal), and GAPDH (ab8245, Abcam). FITC-conjugated secondary antibodies were purchased from Millipore. DHA (HY-B2167), EPA (HY-B0660), corn oil (HY-Y1888), ferrostatin-1 (Fer-1, HY-100579), liproxstatin-1 (Lip-1, HY-12726), Z-VAD-FMK (Z-VAD, HY-16658B), necrostatin-1 (Ne-1, HY-15760), H-89 (HY-15979), Zileuton (HY-14164), GLX351322 (HY-100111), FITC-dextran (MW 70000, HY-128868E) were purchased from MedChemExpress. HG106 (E1080) and erastin (S7242) was obtained from Selleck.

### Cell culture

Normal human gastric epithelial cell line (NGEC) and normal human small intestinal epithelial cell line (HIEC) were obtained from Otwo Biotech. Cells were cultured in DMEM supplemented with 10% FBS and 1% penicillin-streptomycin. For normoxic experiments, cells were incubated at 37°C in a humidified incubator containing 5% CO_2_. For hypoxic experiments, cells were exposed to 1% O_2_ and 5% CO_2_ in a hypoxic incubator (GC-C01, NAWORDE).

### Cell treatment

NGEC and HIEC were first exposed to normoxic or hypoxic conditions for 24 h, followed by treatment with dimethyl sulfoxide (DMSO), DHA (1, 10, 25, 50 μM), or EPA (1, 10, 25, 50 μM) for an additional 24 h under the same environmental conditions. After treatment, cells were harvested for crystal violet staining, cell counting kit-8 (CCK-8) assay, trypan blue staining, transmission electron microscopy (TEM), lipidomic analysis, Western blotting, and 4-hydroxynonenal (4-HNE) and malondialdehyde (MDA) quantification.

For the intervention experiments with inhibitors of necrosis, apoptosis, ferroptosis, SLC7A11, PKA, ALOX5, or NOX4, cells were exposed to normoxic or hypoxic conditions for 24 h, followed by incubation with DMSO, DHA (50 μM), or EPA (50 μM), in the presence or absence of Nec-1 (10 μM, 50 μM, or 100 μM), Z-VAD (10 μM, 20 μM, or 50 μM), Fer-1 (5 μM), Lip-1 (1 μM), HG106 (20 μM), H89 (10 μM), or Zileuton (10 μM) plus GLX351322 (10 μM) for an additional 24 h. Cells were then collected for crystal violet staining, CCK-8 assay, trypan blue staining, western bloting, cyclic adenosine monophosphate (cAMP) assay, protein kinase A (PKA) activity assay, and 4-HNE quantification.

For the transfection experiments, NGEC and HIEC were transiently transfected with either an empty pCMV vector (control) or a pCMV-ATF3 plasmid containing full-length wild-type ATF3 cDNA. Twenty-four hours post-transfection, cells were treated with DMSO, DHA (50 μM), or EPA (50 μM) for an additional 24 h and then harvested for Western blot analysis.

For the experiments demonstrating that ferroptosis inhibitors effectively rescue the cell death phenotype induced by ferroptosis agonist, cells were incubated with erastin (50 μM) in the presence or absence of Fer-1 (5 μM) or Lip-1 (1 μM) for 24 h, and cells were then collected for CCK-8 assay and trypan blue staining.

### Animal experiments

Animal experiments were conducted using six-to eight-week-old male and female C57BL/6J mice (Charles River Laboratories) housed in a specific-pathogen-free environment at Tsinghua University. All experimental protocols were approved by the Animal Care and Use Committee of Tsinghua University (protocol #23-CZJ1), and all methods were performed in accordance with the guidelines. Mice were group-housed (five per cage) in standard plastic cages and randomly assigned to four experimental groups (n = 10 per group) based on gender and age. For the normoxia control group, mice were maintained under normoxic conditions for 3 days and received oral gavaged of 50 μl corn oil on days 1 and 2. For the hypoxia control group, mice were placed in a hypoxic chamber (Yuyan Instruments) with an oxygen concentration equivalent to 65% of sea level oxygen levels for 3 days, receiving 50 μl corn oil by oral gavage on days 1 and 2. In the DHA or EPA intervention groups, mice were exposed to the same hypoxic conditions for 3 days and administered DHA (50 mg/kg in 50 μl corn oil) or EPA (50 mg/kg in 50 μl corn oil) by oral gavage on days 1 and 2. In the Fer-1 treatment experiment (n = 10 per group, with n = 5 per group for assessment of gastrointestinal permeability), mice were exposed to hypoxia for 3 days and received intraperitoneal injections of either a control solvent (2% DMSO + 98% PBS, 50 μl) or Fer-1 (1 mg/kg body weight, 50 μl) on day 1. Mice exposed to normoxia and administered intraperitoneal injections of control solvent on day 1 served as the normal controls. To assess the severity of hypoxia-induced gastrointestinal injury, daily measurements of body weight, food intake, stool consistency, and the presence of bloody stool were recorded. Stool consistency and bloody stool scores were calculated as previously described (15). On day 3, mice were euthanized, and gastric and small intestinal mucosal tissues were collected for Western blot analysis, lipid hydroperoxide (LPO) and 4-HNE analysis, hematoxylin and eosin (H&E) staining, TEM analysis, interleukin-6 (IL-6) analysis, and assessment of gastrointestinal permeability.

### Crystal violet staining

NGEC and HIEC were seeded in 12-well plates, gently rinsed two to three times with PBS, and fixed with 4% paraformaldehyde for 1 h. Cells were then stained with 0.1% crystal violet solution (C0121; Beyotime Biotechnology, Shanghai, China) for 30 min. After staining, cells were washed three times with PBS and imaged using an Epson V850P scanner (Suwa, Nagano Prefecture, Japan).

### CCK-8 assay

Cell viability was assessed using the CCK-8 assay. NGEC and HIEC were seeded into 96-well plates. Subsequently, 10 μl of CCK-8 solution (CK04; Dojindo Laboratories) was added to each well, and the plates were incubated for 1 h. Absorbance (OD) was measured at 450 nm.

### Trypan blue staining

Cell suspension was mixed with an equal volume of trypan blue solution (C1313S; Beyotime Biotechnology) and incubated for 3–5 min at room temperature. Then the mixture was loaded into a hemocytometer chamber and examined by an automatic cell analyzer (Countstar Mira BF; Ruiyu Biotech). The rate of cell death was calculated as the ratio of trypan blue-positive cells to total cells.

### H&E staining

Fresh gastric and small intestinal tissues were fixed in 4% paraformaldehyde, embedded in paraffin, and sectioned into 5-μm-thick slices. Tissue sections were stained with H&E to evaluate histological changes. Histological scoring of the stomach and small intestine was performed following a previously established protocol (18).

### TEM analysis

Ultrastructural changes in gastric and small intestinal tissues, as well as in NGEC and HIEC, were analyzed using TEM. Briefly, tissue samples (1 mm^3^) and cultured cells were fixed in 2.5% glutaraldehyde, followed by secondary fixation with 1% osmium tetroxide, gradient dehydration, permeation, embedding, sectioning (80–100 nm thick), and staining. Processed samples were visualized using a TECNAI G 20 TWIN TEM (FEI Company, Hillsboro).

### Assessment of gastrointestinal permeability

To determine gastrointestinal permeability in the Fer-1 treatment experiment, mice were fasted for 4 h before being orally gavaged with FITC-dextran (MV 70,000; 0.6 mg/g body weight) on day 3. Blood samples were collected 4 h after administration, and serum FITC-dextran concentrations were obtained using fluorometry at 520 nm wavelength.

### Western blot analysis

Total protein was extracted from NGEC and HIEC using RIPA lysis buffer (P0013 B; Beyotime) and quantified using the Pierce BCA Protein Assay Kit (Thermo Fisher Scientific). Equal amounts of protein were separated by 12% SDS-PAGE and transferred onto nitrocellulose membranes. Membranes were blocked with 5% skimmed milk for 2 h and incubated with primary antibodies overnight at 4°C. After 3 washes with TBST, membranes were incubated with secondary antibodies at room temperature for 2 h. Protein bands were visualized using a gel imaging system (SinSage Technology Co., Ltd).

### Enzyme-linked immunosorbent assay (ELISA)

The levels of cAMP and PKA activity in cell lysates, as well as IL-6 expression in murine tissues, were determined by using a cAMP kit (RK04812; Abcolonal), a PKA activity Kit (ab139435; Abcam), and a mouse IL-6 Elisa kit (ab222503; Abcam), respectively, according to the manufacturer’s protocols. The OD was measured at a wavelength of 450 nm.

### Assays for the measurement of lipid peroxidation

Lipid peroxidation levels were assessed using 4-HNE (E-EL-0128c), MDA (E-BC-K028-M), and LPO (E-BC-K176-M) assay kits (all from Elabscience) according to the manufacturer’s instructions.

### Lipidomic analysis

Lipid extraction and lipidomic profiling of HIEC were performed following a previously established protocol (15).

### Statistical analysis

Statistical analyses were performed using GraphPad Prism 10. Data were expressed as mean ± standard error of the mean (SEM) for all in vivo and in vitro experiments. Comparisons between two groups were performed using independent sample *t* tests, while comparisons among three or more groups were analyzed using one or two-way analysis of variance (ANOVA) with post hoc Bonferroni correction. A *P* value < 0.05 was considered statistically significant.

## Results

### Ferroptosis inhibitors attenuated hypoxia-induced cell death exacerbated by DHA and EPA in NGEC and HIEC

**Fig. 1:**
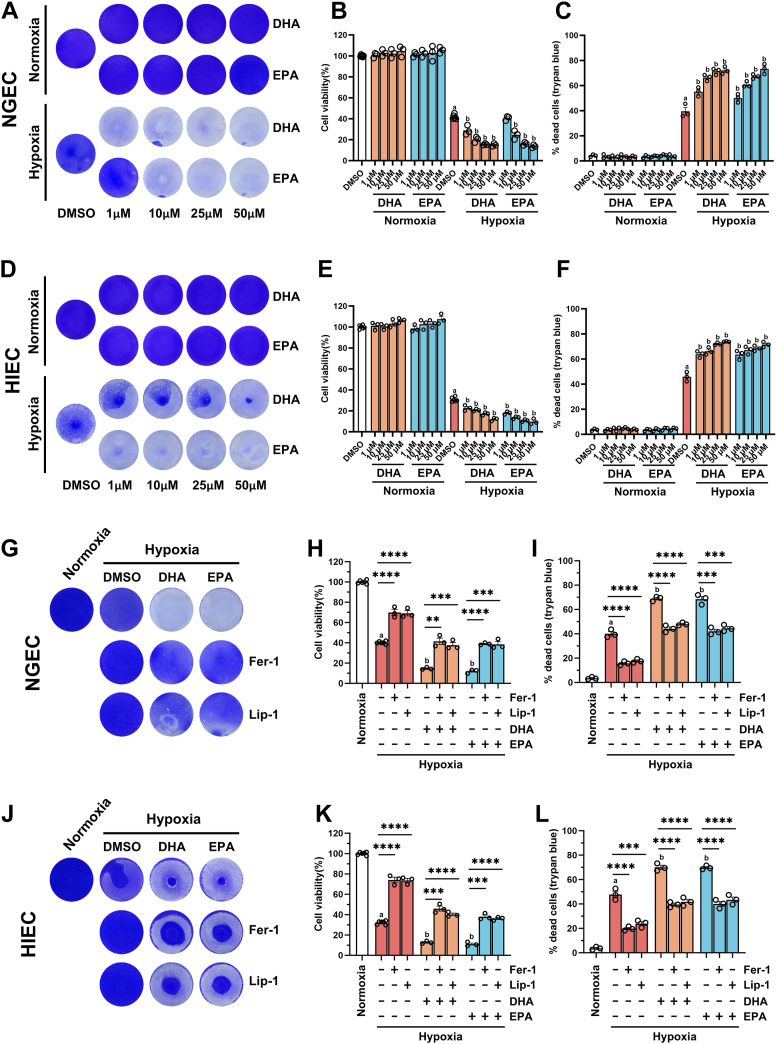
Ferroptosis inhibitors attenuated hypoxia-induced cell death in NGEC and HIEC exacerbated by DHA and EPA supplementation. NGEC and HIEC were cultured under hypoxia (1% O_2_) or normoxia for 24 h, followed by treatment with dimethyl sulfoxide (DMSO), docosahexaenoic acid (DHA; 1, 10, 25, 50 μM), or eicosapentaenoic acid (EPA; 1, 10, 25, 50 μM) for an additional 24 h under the same oxygen conditions. Crystal violet staining of NGEC (A) and HIEC (D). Cell viability of NGEC (B) and HIEC (E) was evaluated by CCK-8 assay. Cell death rate of NGEC (C) and HIEC (F) assessed by trypan blue staining. After 24 h of hypoxia or normoxia, NGEC and HIEC were treated with DMSO, DHA (50 μM), EPA (50 μM), ferrostatin-1 (Fer-1, 5 μM), or liproxstatin-1 (Lip-1, 1 μM) for an additional 24 h. Crystal violet staining of NGEC (G) and HIEC (J). Cell viability of NGEC (H) and HIEC (K) was evaluated by CCK8 assay. Cell death rate of NGEC (I) and HIEC (L) was analyzed by trypan blue staining. Statistical comparisons: hypoxia control versus normoxia control, ^a^*P* < 0.0001; hypoxia + DHA or hypoxia + EPA versus hypoxia control, ^b^*P* < 0.05 at least, in (B,C, E, F, H, I, K, L). ∗*P* < 0.05, ∗∗*P* < 0.01, ∗∗∗*P* < 0.001, and ∗∗∗∗*P* < 0.0001; ns, not significant. Error bars denote means ± standard errors of the mean (SEM).

To determine the effects of DHA and EPA on the viability of human gastric and small intestinal epithelial cells, NGEC and HIEC were cultured under normoxic and hypoxic conditions. Under normoxia, DHA and EPA treatment exhibited no significant effect on cell viability. However, under hypoxia, a concentration-dependent decrease in cell viability was observed following DHA and EPA treatment in both cell lines (Fig. 1A, B, D, E). To determine whether DHA- and EPA-induced reduction in cell viability resulted from cell death, trypan blue staining was performed. The results revealed that DHA and EPA supplementation under hypoxia significantly exacerbated hypoxia-induced cell death in a concentration-dependent manner (Fig. 1C, F). Based on our previous study demonstrating that ferroptosis plays a crucial role in hypoxia-induced gastric and small intestinal injury (15), we hypothesized that ferroptosis also contributed to DHA- and EPA-induced cell death under hypoxic conditions. To test this hypothesis, cells were treated with ferroptosis inhibitors Fer-1 and Lip-1. Crystal violet staining and CCK-8 assay revealed that Fer-1 and Lip-1 elevated the cell viability decreased by DHA and EPA in NGEC and HIEC under hypoxia (Fig. 1G, H, J, K). As shown by trypan blue staining, Fer-1 and Lip-1 increased cell viability largely by decreasing cell death under hypoxia (Fig. 1I, L). Furthermore, CCK-8 assay, and trypan blue staining were used to validate whether the observed effects were ferroptosis-specific, and we found that Fer-1 and Lip-1 effectively reversed the cell death induced by erastin (supplemental Fig. S3). These findings suggest ferroptosis mediates the exacerbated cell death induced by DHA and EPA in hypoxic conditions (Fig. 1G–L).

### DHA and EPA exacerbated hypoxia-induced ferroptosis in NGEC and HIEC

**Fig. 2:**
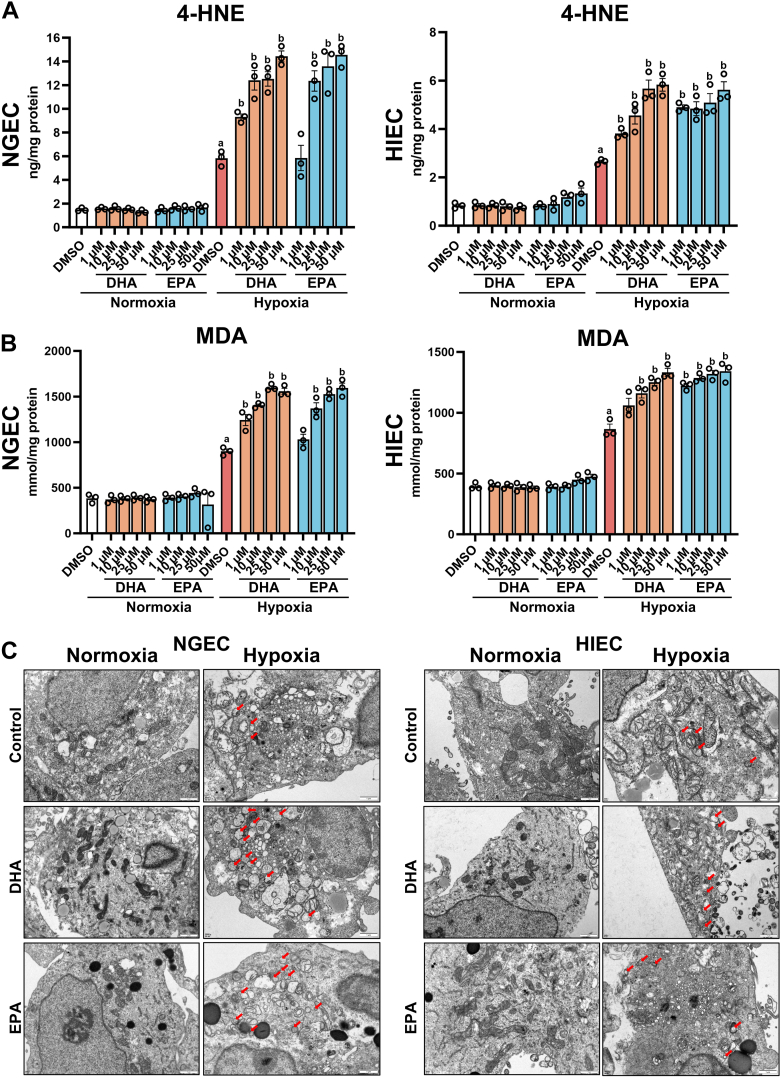
DHA and EPA exacerbated hypoxia-induced ferroptosis in NGEC and HIEC. NGEC and HIEC were cultured under hypoxia (1% O_2_) or normoxia for 24 h, followed by treatment with dimethyl sulfoxide (DMSO), docosahexaenoic acid (DHA, 50 μM), or eicosapentaenoic acid (EPA, 50 μM) for another 24 h under the same oxygen conditions. Quantification of 4-HNE (A) and MDA (B) in NGEC and HIEC. (C) Transmission electron microscopy (TEM) images of NGEC and HIEC (scale bar: 1 μm). Red arrows indicate mitochondria exhibiting ferroptotic characteristics. Statistical comparisons: hypoxia control versus normoxia control, ^a^*P* < 0.0001; hypoxia + DHA or hypoxia + EPA versus hypoxia control, ^b^*P* < 0.05 at least, in (A, B). Error bars denote means ± standard errors of the mean (SEM).

To further confirm the role of ferroptosis in DHA- and EPA-induced cell death under hypoxic conditions, lipid peroxidation in NGEC and HIEC was assessed. The result revealed that under hypoxia, DHA and EPA significantly increased 4-HNE and MDA levels. In contrast, no significant changes were observed among the control, DHA, and EPA groups under normoxia (Fig. 2A–B). Additionally, TEM analysis revealed that DHA and EPA treatment under hypoxia significantly increased the number of mitochondria exhibiting ferroptotic characteristics, including mitochondrial shrinkage, loss of mitochondrial cristae, and increased mitochondrial membrane density. However, these changes were not observed under normoxic conditions (Fig. 2C).

### DHA and EPA upregulated SLC7A11 expression through the cAMP/PKA/ATF3 pathway to protect against self-induced ferroptosis under normoxia

**Fig. 3:**
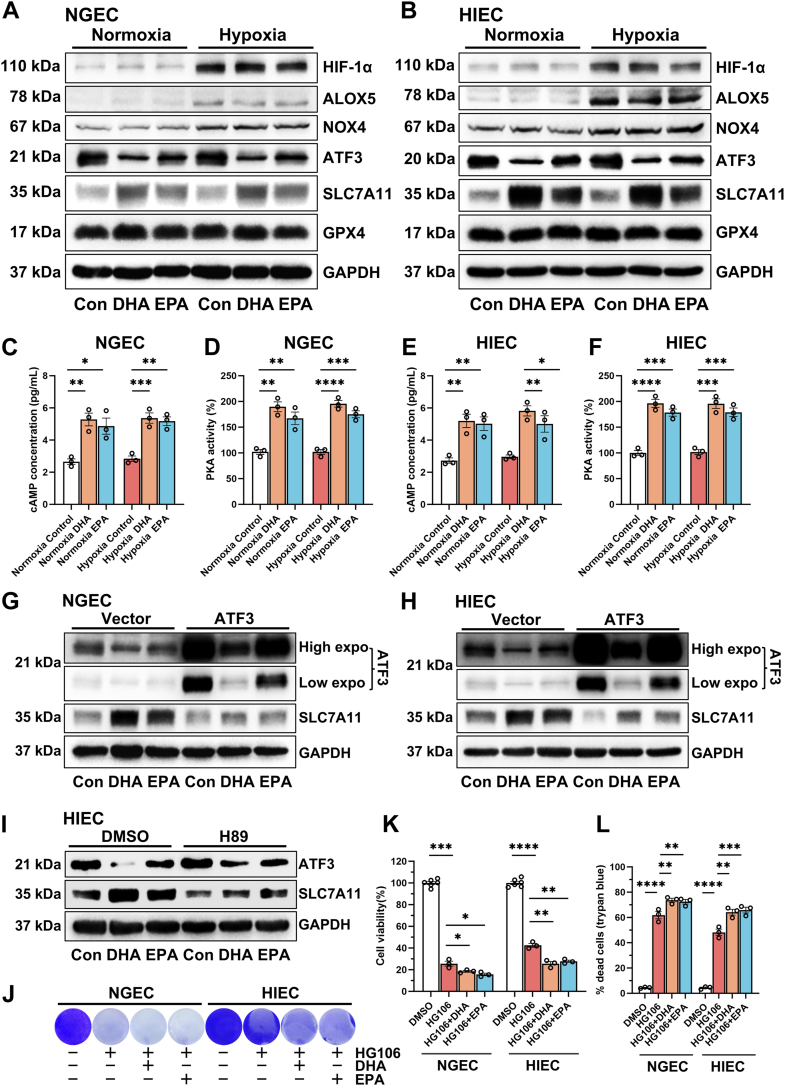
DHA and EPA upregulated SLC7A11 expression through the cAMP/PKA/ATF3 pathway to protect against self-induced ferroptosis under normoxia. NGEC and HIEC were cultured under hypoxia (1% O_2_) or normoxia for 24 h, followed by treatment with dimethyl sulfoxide (DMSO), docosahexaenoic acid (DHA, 50 μM) or eicosapentaenoic acid (EPA, 50 μM) for another 24 h under the same oxygen conditions. Western blot analysis of HIF-1α, ALOX5, NOX4, ATF3, SLC7A11, GPX4, and GAPDH expression in NGEC (A) and HIEC (B). The levels of cyclic adenosine monophosphate (cAMP) and protein kinase A (PKA) activity in NGEC (C, D) and HIEC (E, F) were measured by enzyme-linked immunosorbent assay (ELISA). NGEC and HIEC were transfected with a pCMV vector plasmid or a pCMV-ATF3 plasmid for 24 h, followed by treatment with DMSO, DHA (50 μM), or EPA (50 μM) for another 24 h under normoxia. Western blot analysis of ATF3, SLC7A11, and GAPDH protein levels in NGEC (G) and HIEC (H). High expo in (G, H) indicates high exposure, while low expo indicates low exposure. HIEC was treated with DHA (50 μM) or EPA (50 μM), in the presence or absence of H89 (10 μM, a PKA inhibitor), under normoxic conditions for 24 h. I: The protein levels of ATF3, SLC7A11, and GAPDH by Western blot analysis. NGEC and HIEC were treated with DHA (50 μM) or EPA (50 μM), in the presence or absence of HG106 (20 μM, an SLC7A11 inhibitor), under normoxia for 24 h. J: Crystal violet staining. K: Cell viability analysis using the CCK-8 assay. L: Cell death rate analyzed by trypan blue staining. ∗*P* < 0.05, ∗∗*P* < 0.01, ∗∗∗*P* < 0.001, and ∗∗∗∗*P* < 0.0001; ns, not significant. Error bars denote means ± standard errors of the mean (SEM).

Western blot analysis showed that DHA and EPA upregulated SLC7A11 expression in NGEC and HIEC, regardless of oxygen availability (Fig. 3A). Consistent with our previous findings (15), hypoxia significantly increased the expression of HIF-1α, ALOX5, and NOX4. However, DHA and EPA treatment did not significantly alter the expression of these proteins (Fig. 3A, B). Studies have shown that ATF3 suppresses SLC7A11 expression by binding to the BS-1 and BS-2 sites on the SCL7A11 promoter in a p53-independent manner (19). We thus assessed the expression of ATF3 and found that DHA and EPA inhibited ATF3 expression (Fig. 3A, B). To determine whether DHA- and EPA-induced SLC7A11 upregulation was mediated by ATF3 inhibition, NGEC and HIEC overexpressing ATF3 were treated with DHA and EPA. The Western blot results revealed that ATF3 overexpression significantly downregulated SLC7A11 expression. However, DHA and EPA treatment counteracted this effect by inhibiting ATF3, thereby increasing SLC7A11 levels (Fig. 3G, H). These findings indicate that DHA and EPA promote SLC7A11 expression by downregulating ATF3. Previous studies have found that, DHA and EPA upregulate cAMP/PKA (20), and inhibition of PKA upregulates ATF3 (21). Therefore, we hypothesized that DHA and EPA might enhance the expression of SLC7A11 through the PKA/cAMP/ATF3 pathway. We first analyzed whether DHA and EPA could elevate cAMP levles and PKA activity to validate this hypothesis. The Elisa results showed that DHA and EPA significantly enhanced cAMP levels and PKA activity (Fig. 3C–F). Next, HIEC was treated with DHA, EPA, and the PKA inhibitor H89. Western blot analysis showed that inhibition of PKA attenuated both the DHA- and EPA-induced upregulation of SLC7A11 and the downregulation of ATF3 (Fig. 3I). These findings indicate that DHA and EPA enhance SLC7A11 expression through the PKA/cAMP/ATF3 pathway. After elucidating the regulatory mechanism of DHA and EPA on SLC7A11, we need to clarify the functional significance of SLC7A11 upregulation by DHA/EPA. NGEC and HIEC were treated with high concentrations of the SLC7A11 inhibitor HG106 (20 μM) to ensure that high levels of SLC7A11 expression induced by DHA/EPA were completely suppressed. Crystal violet staining, CCK-8 assay, and trypan staining showed that HG106 induced cell death, which was further exacerbated by DHA or EPA treatment (Fig. 3J–L). These results, together with findings in Fig. 1A–F and Fig. 2, suggest that DHA and EPA protect against self-induced lipid peroxidation by upregulating SLC7A11 expression under normoxia.

### DHA and EPA potentiated hypoxia-induced PUFA-PL accumulation

**Fig. 4:**
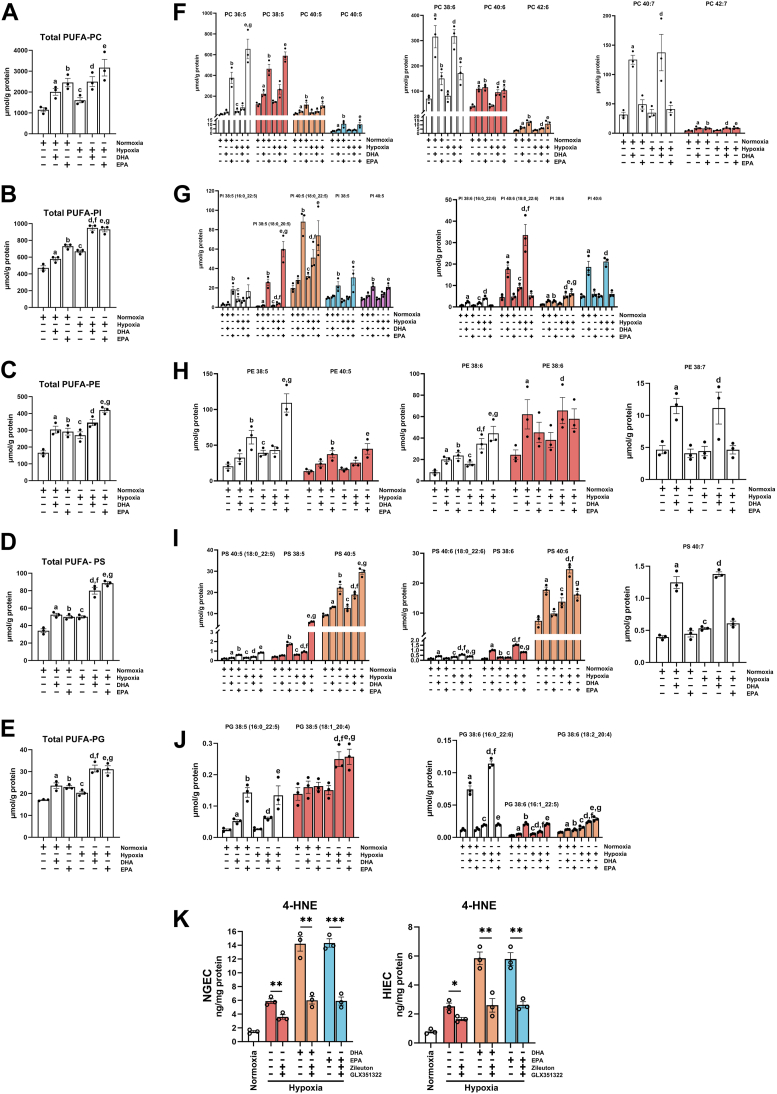
DHA and EPA potentiated hypoxia-induced PUFA-PL accumulation. HIEC was subjected to hypoxia (1% O_2_) or normoxia for 24 h, followed by administration of dimethyl sulfoxide (DMSO), docosahexaenoic acid (DHA) at 50 μM, or eicosapentaenoic acid (EPA) at 50 μM for an additional 24 h under the same oxygen conditions. A–E: The total levels of PUFA-PCs, -PIs, -PEs, -PSs, and -PGs across experimental groups by lipidomic analysis. F–J: The levels of PUFA-PCs, -PIs, -PEs, -PSs, and -PGs with 5–7 carbon-carbon double bonds across experimental groups. NGEC and HIEC were exposed to hypoxic conditions for 24 h, followed by incubation with DHA (50 μM) or EPA (50 μM), in the presence or absence of Zileuton (10 μM) combined with GLX351322 (10 μM) for an additional 24 h. K: The levels of 4-hydroxynonenal (4-HNE) measured by enzyme-linked immunosorbent assay (ELISA). 4-HNE levels in cells under normoxic conditions served as the control. ∗*P* < 0.05, ∗∗*P* < 0.01, ∗∗∗*P* < 0.001, and ∗∗∗∗*P* < 0.0001; ns, not significant. Error bars denote means ± standard errors of the mean (SEM). PUFA, Polyunsaturated fatty acid; PCs, Phosphatidylcholines; PEs, Phosphatidylethanolamines; PGs, Phosphatidylglycerols; PIs, Phosphatidylinositols; PSs, Phosphatidylserines.

Although DHA and EPA exacerbated ferroptosis under hypoxia, no significant differences were observed in SLC7A11 upregulation between normoxic and hypoxic conditions. Therefore, lipidomic analysis was conducted to elucidate the distinct roles of DHA and EPA in PUFA-PLs, including PUFA-phosphatidylcholines (PCs), phosphatidylinositols (PIs), phosphatidylethanolamines (PEs), phosphatidylserines (PSs), and phosphatidylglycerols (PGs). The result revealed that hypoxia, DHA, and EPA significantly increased the levels of PUFA-PCs, -PIs, -PEs, -PSs, and -PGs. Notably, DHA and EPA supplementation under hypoxia resulted in an even greater accumulation of PUFA-PLs than under normoxic conditions (Fig. 4A–E). Since EPA and DHA contain five and six carbon-carbon double bonds, respectively, they can directly form PUFA-PLs with five or more double bonds. To assess the direct influence of DHA and EPA on PUFA-PLs under normoxic and hypoxic conditions, changes in the levels of PCs, PIs, PEs, PSs, and PGs containing five, six and seven carbon-carbon double bonds were analyzed. The results revealed that hypoxia significantly increased the levels of various PUFA-PLs, including PC 36:5, PI 38:5 (16:0_22:5), PI 38:5 (18:0_20:5), PI 40:5 (18:0_22:5), PI 38:6 (16:0_22:6), PI 40:6 (18:0_22:6), PE 38:5, PE 38:6, PS 40:5 (18:0_22:5), PS 38:5, PS 40:5, PS 40:6 (18:0_22:6), PS 38:6, PS 40:6, PS 40:7, PG 38:6 (16:0_22:6), PG 38:6 (16:1_22:5), and PG 38:6 (18:2_20:4). DHA supplementation under normoxia significantly increased the levels of PC 38:5, PC 40:5, PC 42:5, PC 38:6, PC 40:6, PC 42:6, PC 40:7, PC 42:7, PI 38:5 (18:0_20:5), PI 38:6 (16:0_22:6), PI 40:6(18:0_22:6), PI 38:6, PI 40:6, PE 38:6, PE 40:6, PE 38:7, PS 40:5 (18:0_22:5), PS 40:5, PS 40:6 (18:0_22:6), PS 38:6, PS 40:6, PS 40:7, PG 38:5 (16:0_22:5), PG 38:6 (16:0_22:6), PG 38:6 (16:1_22:5), and PG 38:6 (18:2_20:4). Under hypoxia, DHA significantly elevated the levels of PC 40:6, PC 42:6, PC 40:7, PC 42:7, PI 38:5 (18:0_20:5), PI 40:5 (18:0_22:5), PI 38:6 (16:0_22:6), PI 40:6 (18:0_22:6), PI 38:6, PI 40:6, PE 38:6, PE 40:6, PE 38:7, PS 40:5 (18:0_22:5), PS 38:5, PS 40:5, PS 40:6 (18:0_22:6), PS 38:6, PS 40:6, PS 40:7, PG 38:5 (16:0_22:5), PG 38:5 (18:1_20:4), PG 38:6 (16:0_22:6), PG 38:6 (16:1_22:5), and PG 38:6 (18:2_20:4). Notably, DHA supplementation under hypoxia resulted in significantly higher levels of PI 38:5 (18:0_20:5), PI 40:5 (18:0_22:5), PI 40:6 (18:0_22:6), PE 38:6, PS 38:5, PS 40:5, PS 40:6 (18:0_22:6), PS 38:6, PS 40:6, PG 38:5 (18:1_20:4), PG 38:6 (16:0_22:6), PG 38:6 (16:1_22:5), and PG 38:6 (18:2_20:4) than normoxic conditions. This increase in PUFA-PLs potentially contributed to the exacerbation of hypoxia-induced ferroptosis following DHA supplementation. EPA supplementation under normoxia significantly increased the levels of PC 36:5, PC 38:5, PC 40:5, PC 42:5, PC 38:6, PC 40:6, PC 42:6, PC 42:7, PI 38:5 (16:0_22:5), PI 38:5 (18:0_20:5), PI 40:5 (18:0_22:5), PI 38:5, PI 40:5, PI 38:6, PE 38:5, PE 40:5, PE 38:6, PS 40:5 (18:0_22:5), PS 38:5, PS 40:5, PS 38:6, PG 38:5 (16:0_22:5), PG 38:6 (16:1_22:5), and PG 38:6 (18:2_20:4). Under hypoxia, EPA significantly elevated the levels of PC 36:5, PI 38:5 (18:0_20:5), PI 38:6, PE 38:5, PE 38:6, PS 38:5, PS 40:5, PS 40:6 (18:0_22:6), PS 38:6, PS 40:6, PG 38:5 (18:1_20:4), and PG 38:6 (18:2_20:4). Notably, EPA supplementation under hypoxia resulted in significantly higher levels of PC 36:5, PI 38:6, PE 38:5, PE 38:6, PS 38:5, PS 40:5, PS 40:6 (18:0_22:6), PS 38:6, PS 40:6, PG 38:5 (18:1_20:4), and PG 38:6 (18:2_20:4) than normoxia (Fig. 4F–J). This increase in PUFA-PLs potentially exacerbated hypoxia-induced ferroptosis in EPA-treated cells. Considering the abundance, intergroup differences, and carbon-carbon double bonds of DHA (six double bonds) and EPA (five double bonds), we propose that, PI 40:6 (18:0_22:6), PE 38:6, and PS 40:6 are associated with the direct conversion of DHA, and PC 36:5, PI 38:5 (18:0_20:5), PE 38:5, PE 38:6, and PS 40:5 are associated with the direct conversion of EPA.

**Fig. 5:**
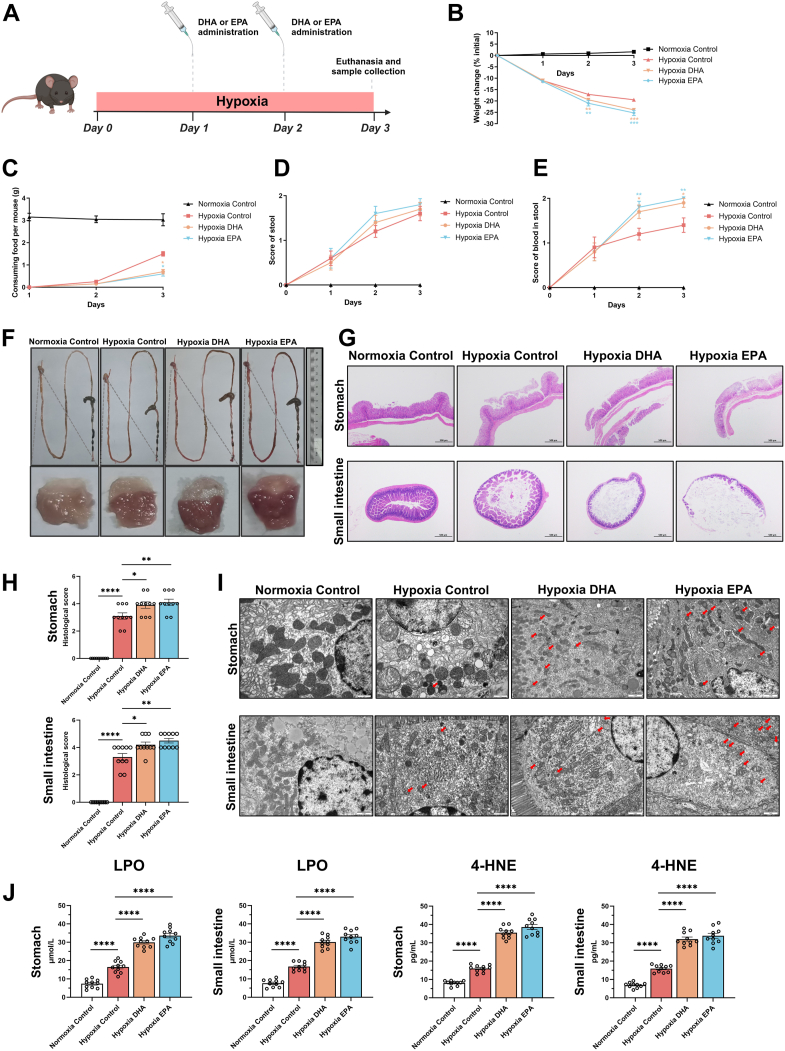
DHA and EPA exacerbated hypoxia-induced ferroptosis in gastric and small intestinal mucosa in vivo. Mice were housed in a hypoxic chamber for 3 days and received oral gavage of corn oil, docosahexaenoic acid (DHA, 50 mg/kg), or eicosapentaenoic acid (EPA, 50 mg/kg) on days 1 and 2. Mice were euthanized on day 3. A: Schematic representation of the experimental protocol. B: Body weight changes. C: Food intake per mouse. D: Stool consistency scores. E: Bloody stool scores. F: Representative images of the gastrointestinal tract and gastric mucosa. G: Representative hematoxylin and eosin (H&E) staining of the stomach and small intestine (40× magnification, scale bar: 500 μm) with corresponding histological scores (H). I: Transmission electron microscopy (TEM) images of the gastric and small intestinal mucosa (scale bar: 1 μm). Red arrows indicate mitochondria exhibiting ferroptotic characteristics. J: Quantification of LPO and 4-HNE levels in the stomach and small intestine. ∗*P* < 0.05, ∗∗*P* < 0.01, ∗∗∗*P* < 0.001, and ∗∗∗∗*P* < 0.0001; ns, not significant. Error bars denote means ± standard errors of the mean (SEM).

Under hypoxic conditions, PUFA-PL levels were increased, and multiple oxidases (among these oxidases, ALOX5 and NOX4 were elevated) directly or indirectly oxidized PUFA-PLs, leading to excessive lipid peroxidation. The administration of DHA/EPA under hypoxia further enhanced PUFA-PL levels, resulting in more severe lipid peroxidation that may obscure the functional significance of enhanced SLC7A11. We thus performed an experiment for this hypothesis. After administering ALOX5 inhibitor (Zileuton) plus NOX4 inhibitor (GLX351322) to suppress certain lipid peroxidation levels, we found that the inhibitory effect of GLX351322 in combination with Zileuton on lipid peroxidation under hypoxia was significantly lower than that observed under hypoxia with the administration of DHA and EPA (Fig. 4K). The results indirectly demonstrate that the elevated SLC7A11 induced by DHA and EPA remains functional under hypoxic conditions.

### DHA and EPA supplementation exacerbated hypoxia-induced ferroptosis in gastric and small intestinal mucosa in vivo

To determine the effects of DHA and EPA on hypoxia-induced ferroptosis in gastric and small intestinal mucosa in vivo, mice were exposed to hypoxia for 3 days and administered DHA or EPA on days 1 and 2 (Fig. 4A). As shown in the Western blot, hypoxia exposure elevated Hif-1α expression in the gastric and small intestinal mucosa of mice, supporting the model validity (Fig. S2). Compared with the hypoxic control group, DHA and EPA supplementation significantly reduced body weight and food intake while exacerbating gastrointestinal symptoms, including bloody and loose stools (Fig. 4B–E). Additionally, DHA and EPA intensified gastrointestinal erythema (Fig. 4F). Histological analysis revealed aggravated mucosal injury in DHA- and EPA-treated mice, characterized by increased epithelial loss and shedding, along with shortened crypts as evidenced by H&E staining and histological scores (Fig. 4G, H). TEM further confirmed mitochondrial alterations in gastric and intestinal mucosal cells, consistent with the in vitro findings (Fig. 4I). Furthermore, DHA and EPA supplementation significantly increased LPO and 4-HNE levels in the gastric and small intestinal mucosa of hypoxic mice (Fig. 4J), reinforcing their role in exacerbating hypoxia-induced ferroptosis.

### Hypoxia plus DHA/EPA-indued cell death in the gastric and intestinal mucosa primarily occurred through ferroptosis rather than necrosis or apoptosis

To determine whether hypoxia-induced cell death in the gastric and intestinal mucosa involved significant apoptosis and necrosis, NGEC and HIEC were treated with necrosis inhibitor (Nec-1) and apoptosis inhibitor (Z-VAD), and the characteristics of apoptosis and necrosis were assessed in the gastric and intestinal mucosa of mice, in the presence or absence of DHA/EPA under hypoxia. The results revealed that Nec-1 and Z-VAD failed to reverse hypoxia-induced cell death in the presence or absence of DHA and EPA (supplemental Fig. S1). Additionally, hypoxia exposure, with or without DHA and EPA, did not significantly elevate the markers of apoptosis and necrosis compared to normoxia (supplemental Fig. S2). To test whether hypoxia-induced gastric and small intestinal mucosal damage was ferroptosis-dependent in vivo, the ferroptosis inhibitor Fer-1 was administered to the mice. Fer-1 significantly mitigated hypoxia-triggered loose and bloody stools, mucosal loss and shedding, 4-HNE in the stomach and small intestine, while exerting a negative impact on the body weight and food intake (supplemental Fig. S4A–C). To clarify the role of Fer-1, gastrointestinal permeability was assessed, and Fer-1 was found to significantly reduce hypoxia-induced hyperpermeability of the gastrointestinal mucosa (supplemental Fig. S4D). Collectively, Fer-1 effectively mitigated hypoxia-induced ferroptosis in the gastric and small intestinal mucosa, but with obvious adverse effects. Therefore, Fer-1 was not used in subsequent in vivo experiments.

The above results indicate that cell death in the gastric and intestinal mucosa induced by hypoxia and by the combination of hypoxia with DHA/EPA primarily occurs through ferroptosis rather than necrosis or apoptosis.

## Discussion

PLs are fundamental components of cellular membranes, forming the lipid bilayers. PUFAs within membrane PLs are highly susceptible to lipid peroxidation, which leads to oxidative membrane damage and ferroptosis (22). DHA and EPA, classified as n-3 PUFA, have been reported to induce or exacerbate ferroptosis. However, studies have also demonstrated that DHA and EPA upregulate SLC7A11, a key inhibitor of ferroptosis (9, 10). In the present study, cell viability and death assays revealed that DHA and EPA did not induce ferroptosis under normoxic conditions but significantly exacerbated hypoxia-induced ferroptosis. Western blot analysis revealed that except SLC7A11, DHA and EPA did not significantly alter the expression of other key ferroptosis-regulating proteins, including ALOX5 and NOX4, under normoxic or hypoxic conditions. Additionally, neither hypoxia nor DHA/EPA supplementation altered the expression of GPX4, ACSL4, LPCAT3, CYPOR, NOX1, and NOX2 (ACSL4, LPCAT3, CYPOR, NOX1, and NOX2 were not shown). Notably, following SLC7A11 inhibition, DHA and EPA further increased cell death under normoxia. This finding suggests that under normoxic conditions, DHA and EPA mitigate their self-induced lipid peroxidation by upregulating SLC7A11. Subsequent experiments further demonstrated that DHA and EPA upregulated SLC7A11 expression through the cAMP/PKA/ATF3 pathway, consistent with previous research demonstrating that DHA and EPA upregulate cAMP/PKA (20), that inhibition of PKA upregulates ATF3 (21), that EPA/DHA-concentrated fish oil inhibits ATF3 (23), and that ATF3 suppresses SLC7A11 expression by binding to the BS-1 and BS-2 sites on the SCL7A11 promoter (19). Our previous study demonstrated that hypoxia induced ferroptosis in gastric and small intestinal epithelial cells by upregulating ALOX5, NOX4, and PUFA-PLs (15). ALOX5 and NOX4 directly or indirectly promote PUFA-PL peroxidation (16, 17). PCs, PIs, PEs, PSs, and PGs are the most commonly studied PLs associated with ferroptosis (24). In this study, lipidomic analysis revealed that hypoxia and DHA/EPA treatment independently increased PUFA-PL levels. Furthermore, hypoxia synergistically enhanced the DHA- and EPA-induced elevation of PUFA-PLs. Notably, DHA increased PUFA-PLs containing six carbon-carbon double bonds, as well as other PUFA-PLs, while EPA similarly elevated PUFA-PLs with five carbon-carbon double bonds along with additional PUFA-PLs. This differential effect may be attributed to the intracellular conversion of DHA and EPA into other PUFAs following cellular uptake. The observed differential effects of DHA and EPA on SLC7A11 expression, PUFA-PL accumulation, and ferroptosis-related phenotypes under normoxic and hypoxic conditions offer mechanistic insights into the distinct roles of these compounds in invalidating ferroptosis under normoxia while significantly potentiating hypoxia-induced ferroptosis. The proposed mechanisms include the following. 1) Under normoxic conditions, the enhanced anti-lipid peroxidation capacity conferred by DHA and EPA, through SLC7A11 upregulation, effectively mitigates the pro-oxidant effects of these compounds induced by increased PUFA-PL levels. 2) Hypoxia induces ferroptosis by upregulating ALOX5, NOX4, and PUFA-PLs. 3) Under hypoxic conditions, DHA and EPA further increase PUFA-PL accumulation, while multiple oxidases (among these oxidases, ALOX5 and NOX4 were elevated) catalyze their peroxidation. These combined effects lead to excessive lipid peroxidation that overwhelms the protective mechanisms mediated by SLC7A11 upregulation, leading to severe ferroptosis. These findings are consistent with our in vivo results, where oral administration of DHA and EPA exacerbated ferroptosis in gastric and small intestinal mucosal cells under hypoxic conditions.

Furthermore, hypoxia has been implicated in inducing ferroptosis of tumors and normal cells in various tissues and organs (15, 25). Therefore, the impact of DHA and EPA on these cells requires further investigation.

## Conclusion

This study demonstrated that DHA and EPA supplementation exacerbates hypoxia-induced ferroptosis in gastric and small intestinal mucosa, resulting in compromised mucosal barrier function. These findings underscore the potential risks of DHA and EPA supplementation under hypoxic conditions, warranting careful evaluation of their intake in individuals at risk of hypoxia.

## Data availability

Data will be made available on request.

## Supplemental data

This article contains supplemental data.

## Conflict of interest

The authors declare that they have no conflicts of interest with the contents of this article.
