# Supplementary material for: DHA and EPA exacerbate hypoxia-induced ferroptosis in gastric and small intestinal mucosa by disrupting the balance between SLC7A11 upregulation and PUFA-PL accumulation
Source: J Lipid Res. 2025 Aug 12;66(9):100876. doi: 10.1016/j.jlr.2025.100876 (PMC12450627; doi:10.1016/j.jlr.2025.100876)
Supplement: Supporting information figures [file mmc1.pptx]

## Slide 1
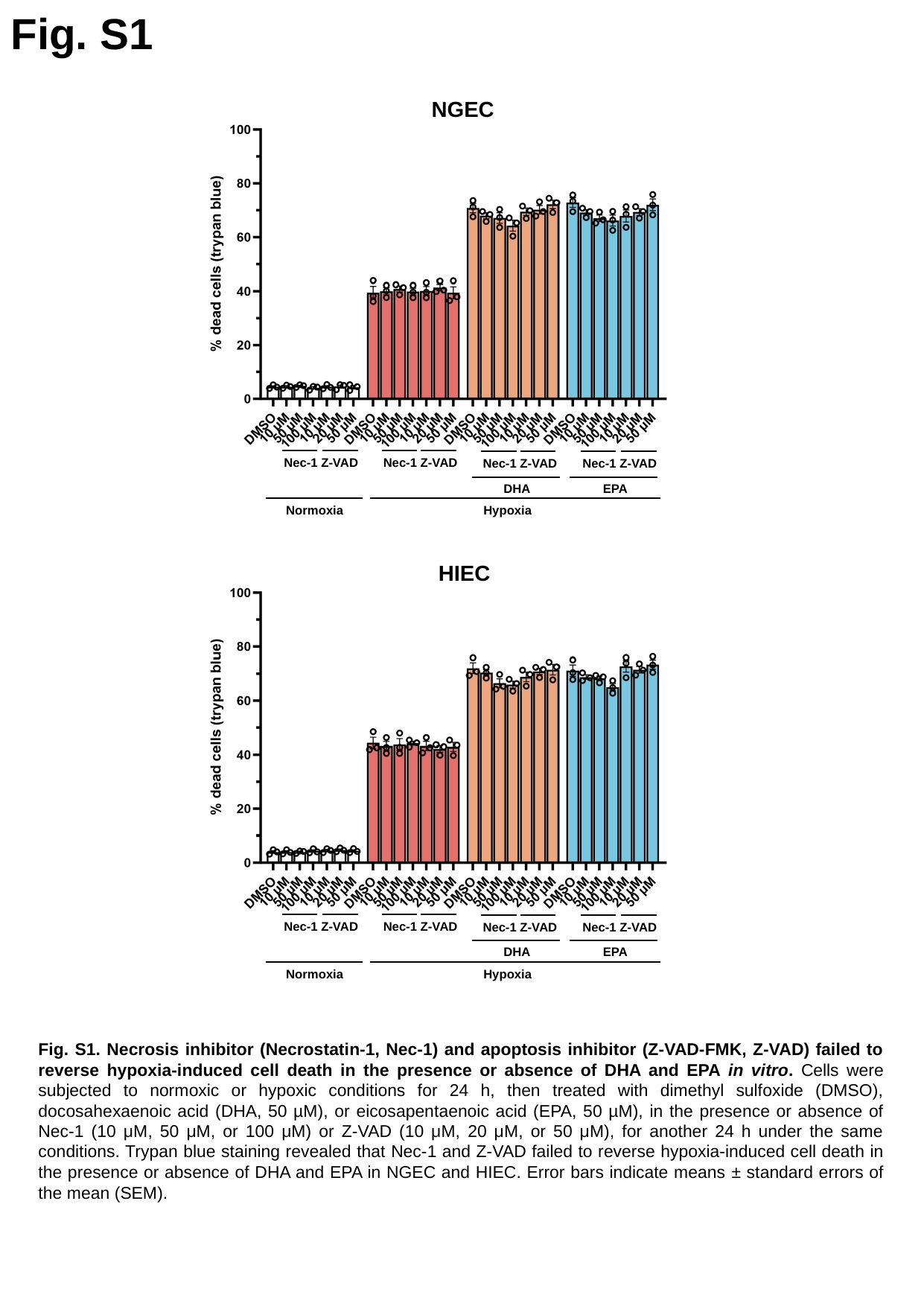

Fig. S1
NGEC
Nec-1 Z-VAD
Nec-1 Z-VAD
Nec-1 Z-VAD
Nec-1 Z-VAD
DHA
EPA
Normoxia
Hypoxia
HIEC
Nec-1 Z-VAD
Nec-1 Z-VAD
Nec-1 Z-VAD
Nec-1 Z-VAD
DHA
EPA
Normoxia
Hypoxia
Fig. S1. Necrosis inhibitor (Necrostatin-1, Nec-1) and apoptosis inhibitor (Z-VAD-FMK, Z-VAD) failed to reverse hypoxia-induced cell death in the presence or absence of DHA and EPA in vitro. Cells were subjected to normoxic or hypoxic conditions for 24 h, then treated with dimethyl sulfoxide (DMSO), docosahexaenoic acid (DHA, 50 µM), or eicosapentaenoic acid (EPA, 50 µM), in the presence or absence of Nec-1 (10 μM, 50 μM, or 100 μM) or Z-VAD (10 μM, 20 μM, or 50 μM), for another 24 h under the same conditions. Trypan blue staining revealed that Nec-1 and Z-VAD failed to reverse hypoxia-induced cell death in the presence or absence of DHA and EPA in NGEC and HIEC. Error bars indicate means ± standard errors of the mean (SEM).

## Slide 2
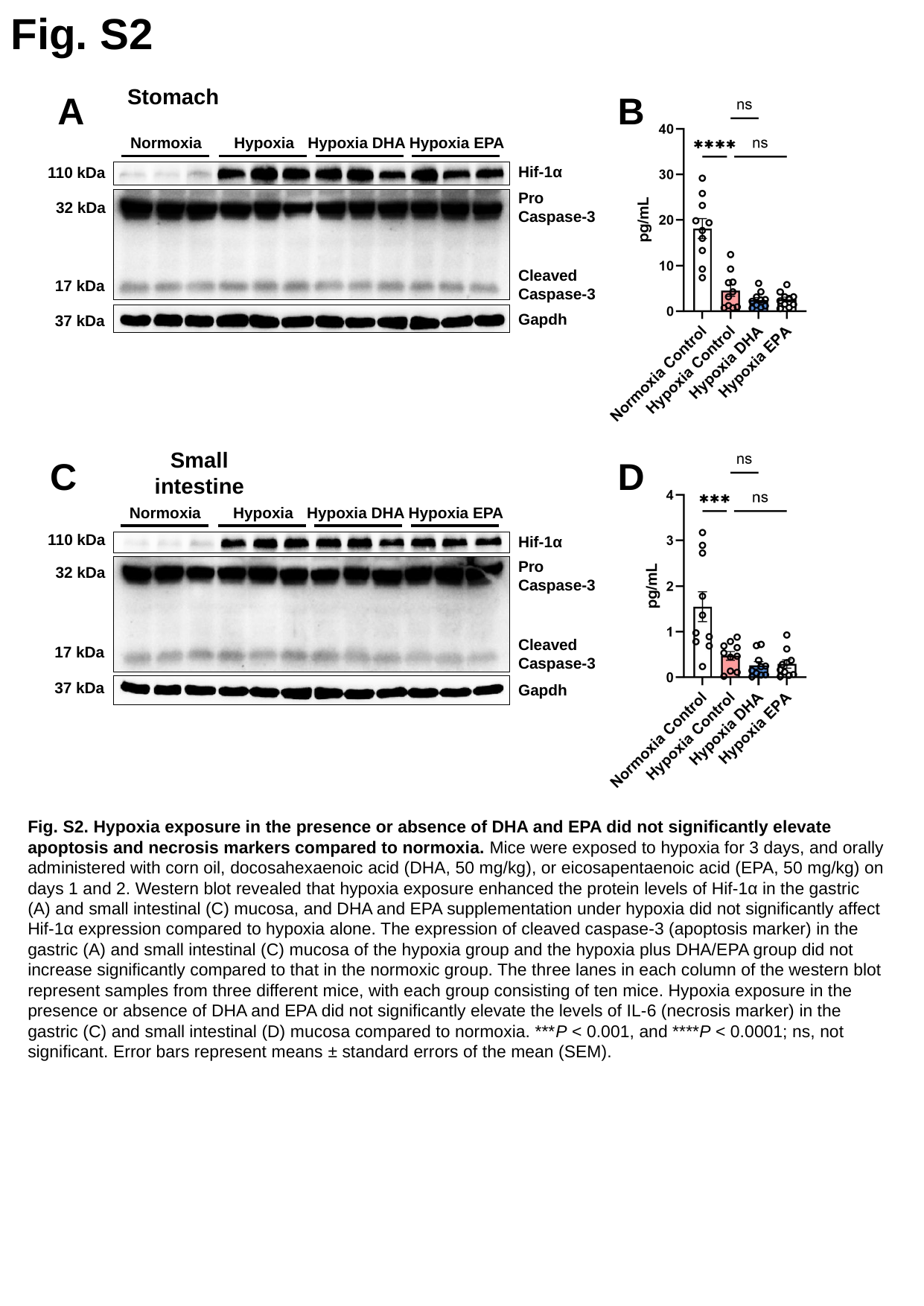

Fig. S2
Stomach
A
B
Normoxia
Hypoxia
Hypoxia DHA
Hypoxia EPA
Hif-1α
110 kDa
Pro
Caspase-3
32 kDa
Cleaved
Caspase-3
17 kDa
Gapdh
37 kDa
Small intestine
C
D
Normoxia
Hypoxia
Hypoxia DHA
Hypoxia EPA
110 kDa
Hif-1α
Pro
Caspase-3
32 kDa
Cleaved
Caspase-3
17 kDa
37 kDa
Gapdh
Fig. S2. Hypoxia exposure in the presence or absence of DHA and EPA did not significantly elevate apoptosis and necrosis markers compared to normoxia. Mice were exposed to hypoxia for 3 days, and orally administered with corn oil, docosahexaenoic acid (DHA, 50 mg/kg), or eicosapentaenoic acid (EPA, 50 mg/kg) on days 1 and 2. Western blot revealed that hypoxia exposure enhanced the protein levels of Hif-1α in the gastric (A) and small intestinal (C) mucosa, and DHA and EPA supplementation under hypoxia did not significantly affect Hif-1α expression compared to hypoxia alone. The expression of cleaved caspase-3 (apoptosis marker) in the gastric (A) and small intestinal (C) mucosa of the hypoxia group and the hypoxia plus DHA/EPA group did not increase significantly compared to that in the normoxic group. The three lanes in each column of the western blot represent samples from three different mice, with each group consisting of ten mice. Hypoxia exposure in the presence or absence of DHA and EPA did not significantly elevate the levels of IL-6 (necrosis marker) in the gastric (C) and small intestinal (D) mucosa compared to normoxia. ***P < 0.001, and ****P < 0.0001; ns, not significant. Error bars represent means ± standard errors of the mean (SEM).

## Slide 3
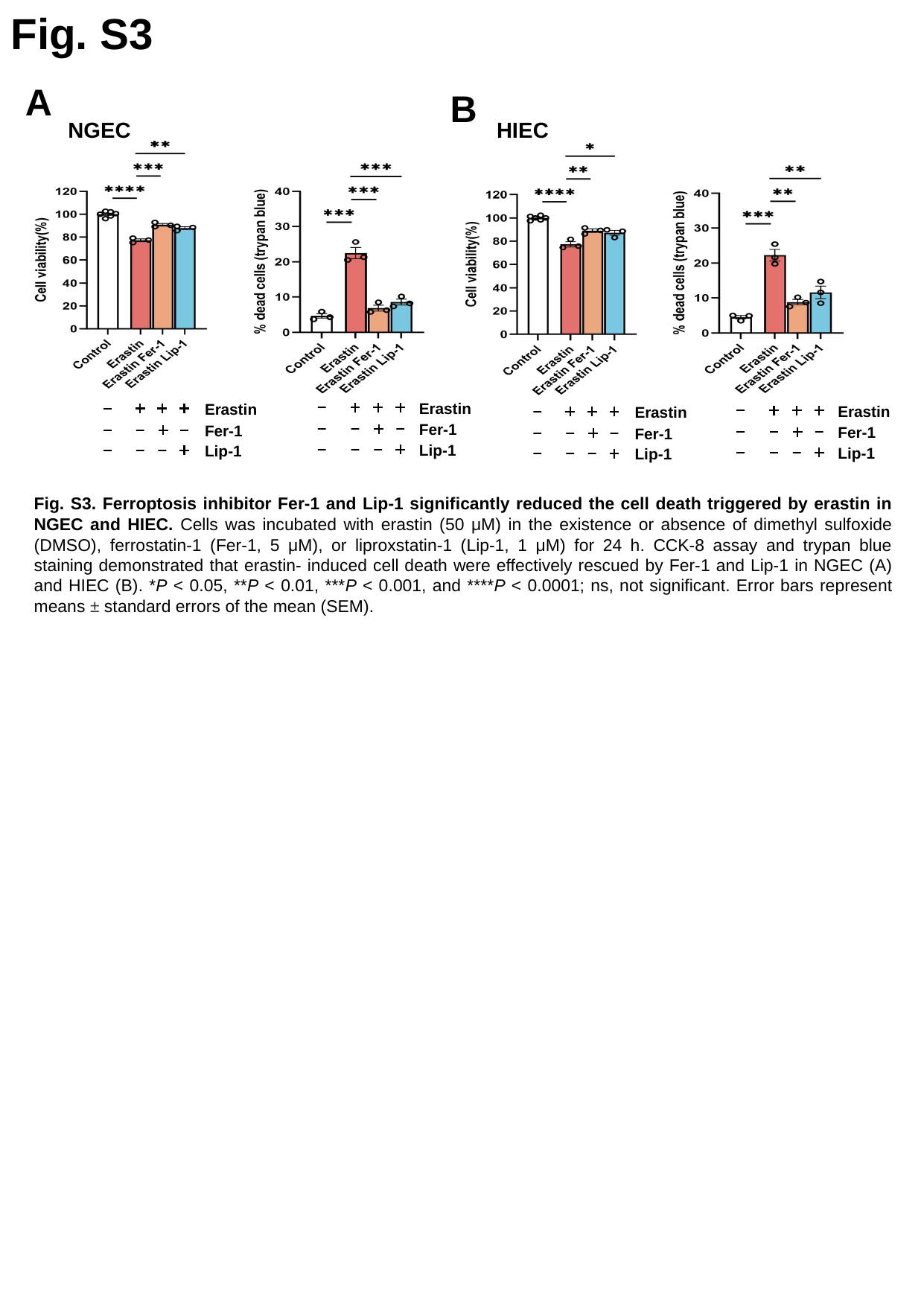

Fig. S3
A
B
NGEC
HIEC
Erastin
Erastin
Erastin
Erastin
Fer-1
Fer-1
Fer-1
Fer-1
Lip-1
Lip-1
Lip-1
Lip-1
Fig. S3. Ferroptosis inhibitor Fer-1 and Lip-1 significantly reduced the cell death triggered by erastin in NGEC and HIEC. Cells was incubated with erastin (50 μM) in the existence or absence of dimethyl sulfoxide (DMSO), ferrostatin-1 (Fer-1, 5 μM), or liproxstatin-1 (Lip-1, 1 μM) for 24 h. CCK-8 assay and trypan blue staining demonstrated that erastin- induced cell death were effectively rescued by Fer-1 and Lip-1 in NGEC (A) and HIEC (B). *P < 0.05, **P < 0.01, ***P < 0.001, and ****P < 0.0001; ns, not significant. Error bars represent means ± standard errors of the mean (SEM).

## Slide 4
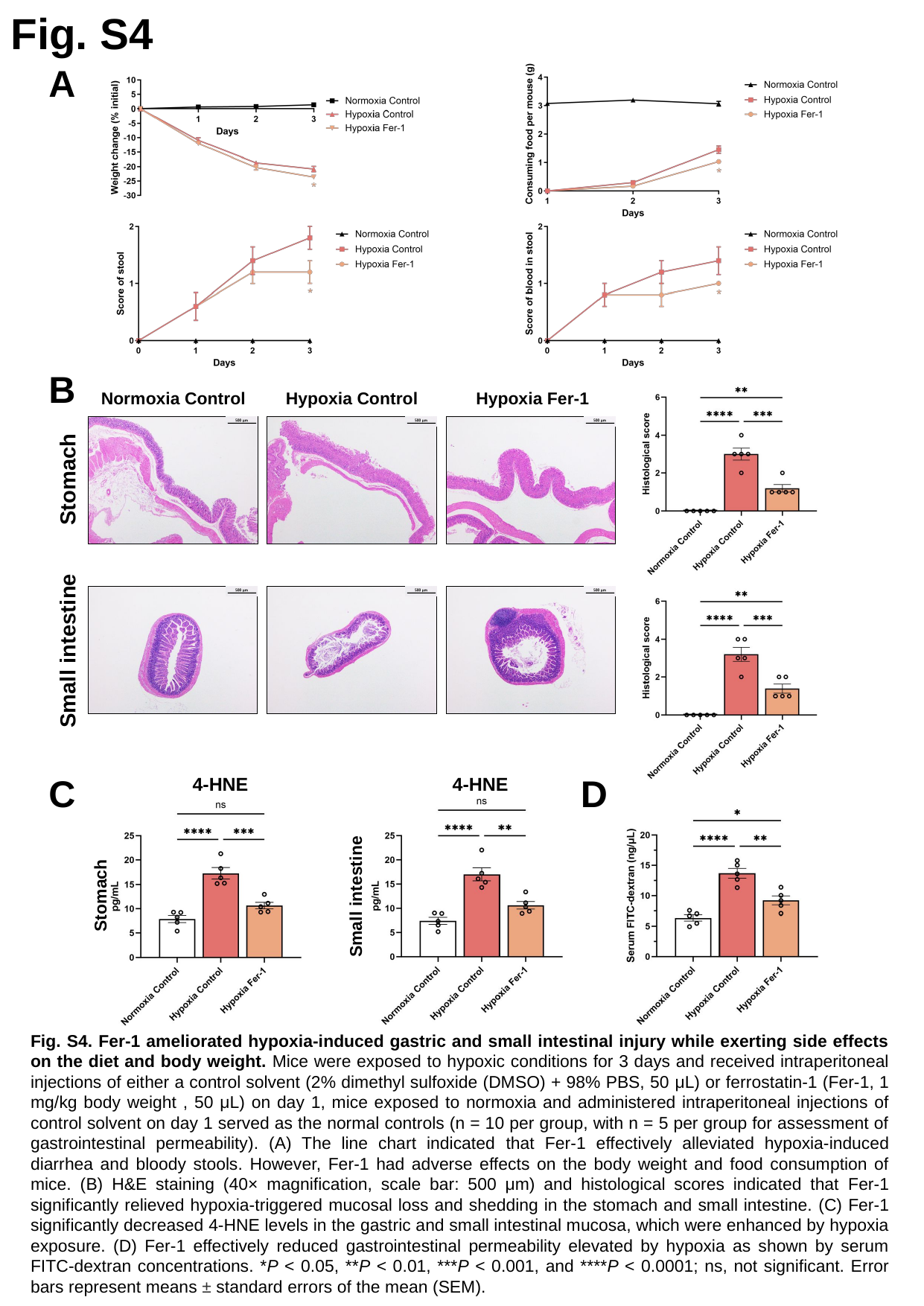

Fig. S4
A
B
Hypoxia Fer-1
Normoxia Control
Hypoxia Control
Stomach
Small intestine
C
D
4-HNE
4-HNE
Stomach
Small intestine
Fig. S4. Fer-1 ameliorated hypoxia-induced gastric and small intestinal injury while exerting side effects on the diet and body weight. Mice were exposed to hypoxic conditions for 3 days and received intraperitoneal injections of either a control solvent (2% dimethyl sulfoxide (DMSO) + 98% PBS, 50 μL) or ferrostatin-1 (Fer-1, 1 mg/kg body weight , 50 μL) on day 1, mice exposed to normoxia and administered intraperitoneal injections of control solvent on day 1 served as the normal controls (n = 10 per group, with n = 5 per group for assessment of gastrointestinal permeability). (A) The line chart indicated that Fer-1 effectively alleviated hypoxia-induced diarrhea and bloody stools. However, Fer-1 had adverse effects on the body weight and food consumption of mice. (B) H&E staining (40× magnification, scale bar: 500 μm) and histological scores indicated that Fer-1 significantly relieved hypoxia-triggered mucosal loss and shedding in the stomach and small intestine. (C) Fer-1 significantly decreased 4-HNE levels in the gastric and small intestinal mucosa, which were enhanced by hypoxia exposure. (D) Fer-1 effectively reduced gastrointestinal permeability elevated by hypoxia as shown by serum FITC-dextran concentrations. *P < 0.05, **P < 0.01, ***P < 0.001, and ****P < 0.0001; ns, not significant. Error bars represent means ± standard errors of the mean (SEM).
